# The *Trypanosoma cruzi* TcrNT2 Nucleoside Transporter Is a Conduit for the Uptake of 5-F-2′-Deoxyuridine and Tubercidin Analogues

**DOI:** 10.3390/molecules27228045

**Published:** 2022-11-19

**Authors:** Mustafa M. Aldfer, Ibrahim A. Alfayez, Hamza A. A. Elati, Nilanjana Gayen, Ehab Kotb Elmahallawy, Ana Milena Murillo, Sabrina Marsiccobetre, Serge Van Calenbergh, Ariel M. Silber, Harry P. de Koning

**Affiliations:** 1 School of Infection and Immunity, College of Medical, Veterinary and Life Sciences, University of Glasgow, Glasgow G12 8TA, UK; 2421701a@student.gla.ac.uk (M.M.A.); ibrahim-fa@hotmail.com (I.A.A.); 2218613e@student.gla.ac.uk (H.A.A.E.); nilanjanagayen57@gmail.com (N.G.); ehab.elmehalawy@vet.sohag.edu.eg (E.K.E.); 2 Department of Zoonoses, Faculty of Veterinary Medicine, Sohag University, Sohag 82524, Egypt; 3 Department of Parasitology, Institute of Biomedical Sciences, University of São Paulo, São Paulo 05508-000, Brazil; ammurillog89@gmail.com (A.M.M.); marsiccobetre@gmail.com (S.M.); ariel.silber@gmail.com (A.M.S.); 4 Laboratory for Medicinal Chemistry (Campus Heymans), Ghent University, B-9000 Gent, Belgium; serge.vancalenbergh@ugent.be

**Keywords:** *Trypanosoma cruzi*, nucleoside transporter, pyrimidine transporter, thymidine, TcrNT2, tubercidin analog, antimetabolite, 5-F-2′-deoxyuridine

## Abstract

Among the scarce validated drug targets against Chagas disease (CD), caused by *Trypanosoma cruzi*, the parasite’s nucleoside salvage system has recently attracted considerable attention. Although the trypanocidal activity of tubercidin (7-deazapurine) has long been known, the identification of a class of 7-substituted tubercidin analogs with potent in vitro and in vivo activity and much-enhanced selectivity has made nucleoside analogs among the most promising lead compounds against CD. Here, we investigate the recently identified TcrNT2 nucleoside transporter and its potential role in antimetabolite chemotherapy. TcrNT2, expressed in a *Leishmania mexicana* cell line lacking the NT1 nucleoside transporter locus, displayed very high selectivity and affinity for thymidine with a K_m_ of 0.26 ± 0.05 µM. The selectivity was explained by interactions of 2-oxo, 4-oxo, 5-Me, 3′-hydroxy and 5′-hydroxy with the transporter binding pocket, whereas a hydroxy group at the 2′ position was deleterious to binding. This made 5-halogenated 2′-deoxyuridine analogues good substrates but 5-F-2′-deoxyuridine displayed disappointing activity against *T. cruzi* trypomastigotes. By comparing the EC_50_ values of tubercidin and its 7-substituted analogues against *L. mexicana* Cas9, Cas9^ΔNT1^ and Cas9^ΔNT1+TcrNT2^ it was shown that TcrNT2 can take up tubercidin and, at a minimum, a subset of the analogs.

## 1. Introduction

*Trypanosoma cruzi*, the etiological agent of Chagas disease (CD), is transmitted principally through the feces/urine of triatomines infected with the protozoan (since triatomines are blood-sucking insects that have the habit of defecating/urinating during or after the blood meals), vertical transmission and oral transfection via infected food items [1,2,3]. The parasite is hard to control, as it infects almost every type of wild and domesticated mammal, in addition to humans [4]. Although there has been some progress in disease control, for instance through better housing and sanitation focused on vector control, the disease appears to be still spreading, and the endemic area has expanded from South America to the Southern United States, both through the spread of the triatomine bugs and through migration of infected individuals [4,5,6]. The life cycle involves epimastigote and metacyclic trypomastigote stages in the bug, as well as bloodstream trypomastigotes and intracellular amastigotes in mammalian hosts [3].

The treatment of CD, caused by the protozoan parasite *T. cruzi*, has not changed for decades despite being highly unsatisfactory [7]. Whereas the acute phase of infection is treated successfully with the old nitro-heterocyclic drugs benznidazole and nifurtimox, neither drug is sufficiently effective to treat the long chronic stage, when most patients are diagnosed, and that can lead to debilitating disease and death [8]. Moreover, for the chronic stage, the drawbacks of the nitro compounds are severe, with treatment duration up to 6 months, leading to severe cumulative toxicity and frequent discontinuation of treatment [9]. Yet, the pipeline of drug discovery for CD remains stubbornly low, with very few new chemical entities at any stage of clinical development in the last decade [7].

One class of potential antiprotozoal compounds that is rapidly gaining attention are nucleoside analogues, which are particularly attractive because none of the protozoan parasites studied is able to make purine nucleosides or nucleobases de novo, and some are also unable to synthesize pyrimidines [10]. It has been known for a long time that some nucleoside antibiotics like tubercidin and cordycepin possess strong activity against kinetoplastid protozoa [11,12,13] but these compounds were either too toxic or not metabolically stable in the mammalian host. More recently, systematic efforts have been made to identify analogs of these antibiotics with improved drug-like properties. For example, cordycepin is rapidly degraded in human plasma by adenosine deaminase [12,14] but it was found that fluorination on the 2-position of the purine ring was protective against this reaction while retaining most of its antitrypanosomal efficacy [15]. Similarly, the introduction of substitutions on position 7 of tubercidin improved activity against a range of protozoa [16,17,18] including *T. cruzi* [16,19,20]. Susceptibility of *T. cruzi* to cordycepin was first reported in 1972 [21].

The efficacy of such compounds is dependent on nucleoside transporters to gain access to the cell and differences in substrate profile of parasite and host carriers may contribute to the selectivity of the compounds [22,23]. To name a few examples, in *Leishmania donovani*, uptake of tubercidin is dependent on nucleoside transporter NT1 [13], and that of formycin B is dependent on NT2 [24]; in *Toxoplasma gondii* uptake of adenine arabinoside is linked to the TgAT1 carrier [25]; in *Trypanosoma brucei*, cordycepin is taken up by the P2/TbAT1 aminopurine transporter [26]), while formycin A and B are taken up by the P1 transporter [27]. Tubercidin, although mostly dependent on P2 for uptake [26] is also taken up by the P1 transport activity [28]. Importantly, some 7-substituted tubercidins such as 7-(2-pyridyl)-tubercidin [16] or 7-Br-tubercdin [17] were found to have approximately equally high affinity (K_i_ < 5 µM) for both transporters and deletion of one of them did not engender resistance. 

In *T. cruzi*, much less is known about nucleoside drug uptake. In 1988, Finley et al. [29] reported that thymidine uptake was affected in tubercidin-resistant *T. cruzi* epimastigotes and we recently reported the cloning of a *T. cruzi* thymidine transporter, TcrNT2 [30]. Although we characterized all four *T. cruzi* Equilibrative Nucleoside Transporter (ENT) family genes, no separate high-affinity adenosine transporter was identified [30,31] and this makes the TcrNT2 transporter the most likely candidate to mediate the uptake of tubercidin and its analogues in this species. Here, we present an in-depth characterization of this carrier including a model of how it interacts with its main substrate, thymidine, and the basis of its selectivity over other pyrimidine nucleosides. We also show that TcrNT2 is an efficient carrier of the cytotoxic thymidine analogue 5-F-2′-deoxyuridine and confirm that it is able to take up tubercidin and a range of tubercidin analogs. The effects of 5-fluorinated pyrimidines against *T. cruzi* epimastigotes and trypomastigotes were also investigated.

## 2. Results

### 2.1. Construction of a ΔNT1 Cell Line of Leishmania Mexicana Promastigotes

In order to eliminate the endogenous transport of adenosine and pyrimidine nucleosides in *L. mexicana,* mediated by LmexNT1, we knocked out the cluster of two *tandem* arrayed *NT1.1* and *NT1.2* genes encoding the LmexNT1 transporter on chromosome 15. For this, we used *L. mexicana* Cas9^ΔNT1^ cells that have been developed for gene editing with the CRISPR-Cas9 system [32,33]. The two genes, LmxM.15.1230.1 and LmxM.15.1240.1, are 99.0% identical by nucleotide and 97.2% by protein sequence (Supplemental Figure S1). 

Briefly, the 5′-sgRNA-NT1 and 3′-sgRNA-NT1 for the deletion of the *NT1* locus were amplified using HDK1508 and HDK1510 as forward primers, respectively, and HDK1502 as reverse primer, serving as template (all primer sequences in Supplemental Table S1). 5′-sgRNA-NT1 and 3′-sgRNA-NT1 were amplified to direct Cas9 to cut immediately upstream (5′) or downstream (3′) of the target locus of *NT1*. The presence of the expected product (~120 bp for both 5′ sgRNA-NT1 and 3′ sgRNA-NT1) was confirmed on a 2% agarose gel (Supplemental Figure S2A). To create CRISPR plasmids specific to the target locus of *NT1*, pTBlast-NT1 and pTPuro-NT1, encoding blasticidin and puromycin resistance markers, were amplified using HDK1507 as forward primer and HDK1509 as reverse primer. The presence of the expected products (~1.7 kb for pTBlast-NT1 and ~1.8 kb for pTPuro-NT1) were visualized on a 1% agarose gel (Supplemental Figure S2B). The deletion of the gene was confirmed by diagnostic PCR with internal primers HDK1523 and HDK1524, specific to the NT1 open reading frame (Supplemental Figure S3) and qRT-PCR (Supplemental Figure S4), showing the absence of the gene and of mRNA, respectively. The ΔNT1 cell line was also confirmed to be resistant to tubercidin, as previously reported [13,34]. Cas9^ΔNT1^ was 60-fold resistant to this adenosine analogue, with an EC_50_ of 25.9 ± 1.9 µM compared to 0.43 ± 0.15 µM for the control *L. mexicana* Cas9 line (*n* = 4; *p* < 0.0001). 

### 2.2. Transport Phenotype of Cas9^ΔNT1^

**Figure 1:**
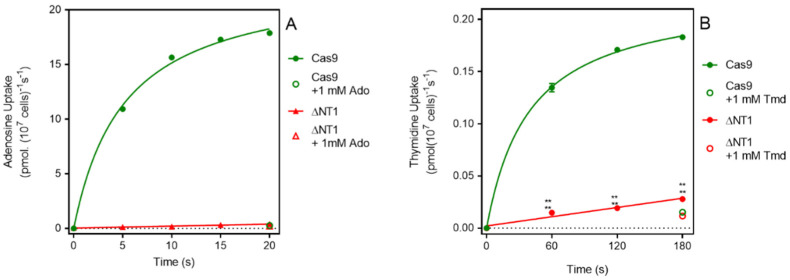
[^3^H]-Adenosine and [^3^H]-Thymidine transport by *L. mexicana* Cas9 and *L. mexicana* Cas9^ΔNT1^. (**A**). Transport of 0.1 µM of [^3^H]-adenosine by *L. mexicana* Cas9 and *L. mexicana* Cas9^ΔNT1^ was measured over 20 s in the presence or absence of 1 mM unlabeled adenosine. (**B**). Transport of 0.05 μM of [^3^H]-thymidine by *L. mexicana* Cas9 and *L. mexicana* Cas9^ΔNT1^ was measured over 180 s in the presence or absence of 1 mM unlabeled thymidine, respectively. Symbols represent the average of triplicate determinations in a single representative experiment and error bars represent ± SEM.

It is well documented that the NT1 transport activity is responsible for the uptake of adenosine and of pyrimidine nucleosides in *Leishmania* spp. [13,35]. In *L. mexicana* the K_m_ values for adenosine and thymidine are 0.81 ± 0.16 µM and 11.2 ± 2.4 µM, respectively [36]. We thus assessed the uptake of these two nucleosides in Cas9^ΔNT1^. Figure 1A shows that the uptake of 0.1 µM [^3^H]-adenosine by *L. mexicana* Cas9 promastigotes was very rapid and became non-linear within a matter of a few seconds, with metabolism apparently limiting the rate of uptake. In contrast, the transport of 0.1 µM of [^3^H]-adenosine by *L. mexicana* Cas9^ΔNT1^ promastigotes was virtually abolished and became statistically identical to the uptake in the presence of 1 mM unlabeled adenosine (*p* > 0.05). We similarly found that the transport of 0.05 µM of [^3^H]-thymidine was greatly reduced in Cas9^ΔNT1^ cells compared with *L. mexicana* Cas9, with a rate of just 0.00004 ± 0.000006 pmol(10^7^ cells)^−1^ s^−1^ (Figure 1B), which nonetheless was still significantly different from zero (F-test, *p* = 0.026), and higher than the saturation control with 1 mM unlabeled thymidine. The abolition of adenosine transport and the near-complete reduction in thymidine transport shows the successful deletion of the NT1 activity. 

### 2.3. Heterologous Expression of TcrNT2 in L. mexicana Cas9^ΔNT1^

In a phylogenetic analysis of *T. cruzi* ENT transporters with other trypanosomatids (*Leishmania* spp., *T. brucei* and *T. congolense*), TcrNT2 (TcCLB.506445.110) is grouped with the *Leishmania* NT1 transporter, which transports pyrimidine nucleosides and adenosine [30]; TcrNT2 displays 50.0% identity with LmexNT1.1 by amino acid sequence. By expressing *TcrNT2* in a *T. b. brucei* cell line lacking nucleobase transporters [37], it was established that the gene encodes a thymidine-specific carrier with a K_m_ of ~0.2 µM, 300-fold lower affinity for uridine and virtually no affinity for cytidine, pyrimidine nucleobases, adenosine or other purines [30]. The *T. brucei* expression worked well enough for the basic characterization of the transporter but these cells still express a multitude of nucleoside transporters [10,38] including for uridine [37]. In contrast, LmexNT2, the sole nucleoside transporter expressed in *L. mexicana* Cas9^ΔNT1^ cells, only has affinity for oxopurine nucleosides [36,39]. We thus resolved to express *TcrNT2* in the Cas9^ΔNT1^ strain in order to explore its utility for the uptake of cytotoxic nucleoside analogues. 

TcrNT2 was amplified by PCR from the plasmid pHD1336 that had served to express it in *T. brucei* [30], using primers HDK 1551 and HDK1552, which introduced *Bgl*II and *Xho*I restriction sites, respectively. The plasmid pNUS-HcN [40] was digested with these enzymes and TcrNT2 was ligated into it to create plasmid pHDK270. After transfection, selection on the antibiotic G418 and cloning by limited dilution, the presence of the gene in selected clones was confirmed by PCR, with TcrNT2-specific primer HDK1551 and HDK340 as reverse primer from the 3′ vector sequence (Supplemental Figure S5). The resulting cell line displayed a similar growth rate as the *L*. *mexicana* Cas9 and Cas9^ΔNT1^ strain albeit consistently slightly slower, with a doubling time of ~6.5 h for each strain (Supplemental Figure S6).

### 2.4. TcrNT2 Is a Conduit for Cytotoxic Uridine and Tubercidin Analogues

As a thymidine transporter, it would be expected that TcrNT2 might similarly transport structural analogues like 5-F-2′-deoxyuridine. Table 1 shows that the Cas9^ΔNT1^ strain was 26.6-fold resistant to this nucleoside but that this was almost completely reversed upon expression of TcrNT2, showing it is indeed a substrate. 5-F-uridine displayed a poor effect on any of the strains (>500 μM), and TcrNT2 did not sensitize to it either; 5-F-uracil was likewise poorly active, with no sensitization by TcrNT2 (Supplemental Figure S7). These fluoro-pyrimidines are therefore either not substrates for either the *L. mexicana* or TcrNT2 transporters, or simply not very toxic to the *L. mexicana* promastigotes. 

Apart from its strong antileishmanial activity, tubercidin has long been known to display strong activity against *T. cruzi* in vitro [29] and the EC_50_ was recently reported as 0.34 µM, with several analogues displaying highly similar activities—i.e., an order of magnitude better than benznidazole [16]. Interestingly, Finley et al. [29] also reported that a tubercidin-resistant transporter mutant was deficient in the uptake of tubercidin and thymidine but not adenosine or inosine, leading them to conclude that a *T. cruzi* thymidine transporter is responsible for the internalization of tubercidin. We therefore compared the sensitivity of the three *L. mexicana* strains Cas9, Cas9^ΔNT1^ and Cas9^ΔNT1+TcrNT2^ to a series of tubercidin analogues. 

Tubercidin was 25-fold resistant in Cas9^ΔNT1^ relative to Cas9 and this was completely reversed upon expression of TcrNT2 (Table 1), showing that tubercidin is indeed able to enter through this carrier. We next explored whether substitutions on position 7 are tolerated, as some 7-substituted analogues have been shown to improve anti-protozoal activity and reduce host toxicity [16,17,20,41]. Halogenation (F, Cl, Br, I) on position 7 did not appear to impede uptake via TcrNT2 as the expression reversed the observed resistance in Cas9^ΔNT1^. A 7-CF_3_ analogue, FH6367, did not display reduced activity in Cas9^ΔNT1^ so no conclusion could be reached as to whether TcrNT2 can transport it, i.e., the transport rate into the *Leishmania* promastigotes was not limiting to its efficacy. Resistance to 7-ethynyl analogue FH3143 was only partially reversed but, importantly, 7-(3,4-dichlorophenyl)-tubercidin FH3147 did appear to be a substrate of TcrNT2. This analogue was among the most active tubercidin analogues identified against intracellular *T. cruzi* amastigotes, with an EC_50_ of 0.19 µM [16]. Strikingly, this TcrNT2-dependent activity of FH3147 seems crucially dependent on the substitution pattern of the 7-phenyl ring since the activity loss observed for the unsubstituted phenyl analogue TH1004 was not reversed upon TcrNT2 expression. We tried to explore this further with the 3-F,4-Cl-phenyl analogue FH10714 (which also has very promising activity against *T. cruzi* [41]) and the 4-Cl-phenyl pyrazolopyrimidine JB602, but neither analogue displayed activity against the *L. mexicana* strains, whether TcrNT2 was expressed in them or not. 

The corresponding 3′-deoxytubercidin analogues appeared to be poorer substrates as the expression of TcrNT2 did not significantly reverse resistance in Cas9^ΔNT1^ to 7-F-3′-deoxytubercidin FH8517 (*p* > 0.05, Table 1). The corresponding 7-Br (FH7429-up) and 7-ethynyl (FH8505) analog displayed somewhat lower EC_50_ values in the Cas9^ΔNT1+TcrNT2^ strain (*p* < 0.05), the 7-phenyl analog FH8480 was not toxic to any of the strains and the 3,4-dichlorophenyl FH8513 analog also displayed a modest gain in activity upon expression of TcrNT2. We conclude that 3′-deoxy analogues are generally less effective substrates than the corresponding ribofuranoses. Fluorination on the 3′ position may partly remedy this. Although 7-F,3′-F-tubercidin (JB588) also appeared to be a relatively poor substrate, with only a minor reversal of resistance (*p* < 0.05), the anti-leishmanial activity of the corresponding 7-I,3′-F-tubercidin (JB526) was greatly enhanced by expression of TcrNT2 (*p* < 0.001). It thus appears that the 3′-hydroxy could be important for recognition by TcrNT2. In contrast, changes to the 6-amine group did not seem to affect uptake by TcrNT2 as judged by the EC_50_ values. 6-*N*-Methyl and 6-methyl substitutions (CL5564 and CL4501) appeared to be tolerated (Table 1). Finally, pyrazolo[3,4-*d*]pyrimidine nucleosides (JBMAM030 (aminopurinol riboside) and JBMAM021) appeared to be poor substrates for TcrNT2, being unable to reverse the resistance engendered by the knockout of LmexNT1. This is notable given their known activity against *T. cruzi* [42], which must be facilitated by a different transporter.

### 2.5. Structure-Activity Exploration of Inhibitor Recognition by TcrNT2

**Figure 2:**
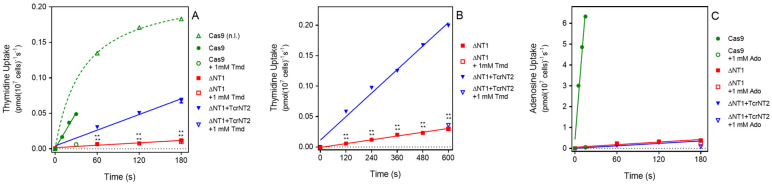
Nucleoside transport by TcrNT2 expressed in *L. mexicana* Cas9^ΔNT1^ (**A**). Transport of 0.05 µM of [^3^H]-thymidine by *L. mexicana* Cas9, *L. mexicana* Cas9^ΔNT1^ and *L. mexicana* Cas9^ΔNT1+TcrNT2^ was measured over 180 s in the presence or absence of 1 mM unlabeled thymidine. (**B**). Transport of 0.05 µM of [^3^H]-thymidine by *L. mexicana* Cas9^ΔNT1^ and *L. mexicana* Cas9^ΔNT1+TcrNT2^ was measured over 10 min in the presence or absence of 1 mM unlabeled thymidine. Symbols represent the average of triplicate determinations in a single representative experiment and error bars represent ± SEM. *p* < 0.001 by F-test. (**C**). Transport of 0.05 µM of [^3^H]-adenosine by *L. mexicana* Cas9, *L. mexicana* Cas9^ΔNT1^ and *L. mexicana* Cas9^ΔNT1+TcrNT2^ was measured over 180 s in the presence or absence of 1 mM unlabeled adenosine. Figure shows a representative experiment in triplicate, and error bars represent ± SEM. F-test was performed using GraphPad Prism 8.

Uptake of 0.05 µM [^3^H]-thymidine was measured over 180 s in all three cell lines. For *L. mexicana* Cas9, uptake over this interval was non-linear, and the experiment was repeated with samples taken at 10, 20 and 30 s only, which yielded a linear phase of uptake (Figure 2A). For Cas9^ΔNT1^ and Cas9^ΔNT1+TcrNT2^ transport was linear over 180 s (Figure 2A) and indeed remained linear for at least 10 min (no significant deviation from linearity, runs test, *p* < 0.4; Figure 2B). The overall rate of uptake in the Cas9^ΔNT1+TcrNT2^ was significantly higher than in the Cas9^ΔNT1^ strain (*p* < 0.0001, F-test), and the uptake at each time point was also significantly higher (*p* < 0.0001, *t*-test, *n* = 3). In contrast, uptake of 0.05 µM [^3^H]-adenosine was not altered upon expression of TcrNT2, with the rate of uptake identical in Cas9^ΔNT1^ and Cas9^ΔNT1+TcrNT2^ (*p* = 0.8, F-test), while the rate of adenosine uptake in both lines was very much lower than in control *L. mexicana* Cas9 cells (*p* < 0.0001; Figure 2C). This establishes that TcrNT2 does not transport adenosine.

**Figure 3:**
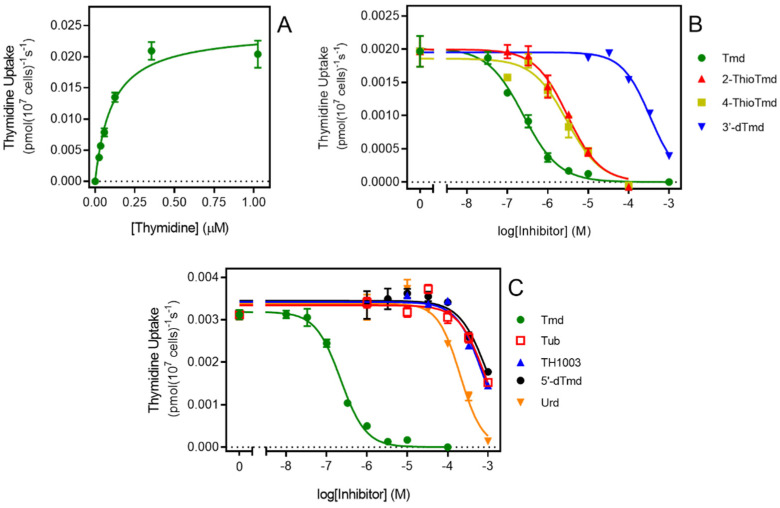
Transport of 0.025 μM [^3^H]-thymidine by TcrNT2. (**A**). Michaelis-Menten plot of thymidine uptake by TcrNT2. Cells were incubated for 5 min with 0.025 μM [^3^H]-thymidine in the presence of variable concentrations of unlabeled thymidine. The experiment is representative of three independent determinations. (**B**,**C**). Dose-dependent inhibition of 0.025 μM thymidine uptake by various unlabeled nucleosides as indicated. Tmd, thymidine; tub, tubercidin; urd, uridine; 5′-dTmd, 5′-deoxythymidine. Error bars are SEM from triplicate determinations.

To gain more insight into how TcrNT2 binds its substrates a series of inhibition experiments was performed using 0.025 µM of [^3^H]-thymidine as the permeant, yielding a series of inhibition constants (K_i_ values), listed in Table 2. These values can be used to calculate the Gibbs free energy of interaction ΔG°, but these experiments cannot distinguish between the test compound being transported or merely binding to the transporter and inhibiting the transit of the [^3^H]-thymidine [43]. We have previously determined the K_m_ of TcrNT2 by expression in *T. brucei* as 0.223 ± 0.007 µM [30]; this was not significantly different from the value obtained in Cas9^ΔNT1+TcrNT2^, 0.263 ± 0.053 µM (*n* = 3; *p* = 0.78, unpaired *t*-test) (Figure 3A). These data indicate that that the heterologous expression is functional and yields consistent results, both internally and in comparison with the expression in *T. b. brucei*.

Figure 3B,C depict inhibition curves with a number of inhibitors tested to probe the structural determinants of high affinity for TcrNT2. One of the most striking features is the almost absolute selectivity for thymidine over uridine (compare 0.26 µM vs. 172 µM for K_i_ value, a difference in ΔG° of 16.1 kJ/mol). Part of this difference is attributable to a positive contribution from the 5-methyl group of thymidine. Pairwise comparisons of the Gibbs Free energy of thymidine vs. 2′-deoxyuridine (δ(ΔG°) = −3.99 kJ/mol) and of 5-methyluridine vs. uridine (δ(ΔG°) = −4.50 kJ/mol) yielded an average interaction energy of 4.2 kJ/mol. This was also consistent with the positive contribution of a 5-I substitution of 4.79 kJ/mol (compare 5-I,2′-deoxyuridine and 2′-deoxyuridine). However, the more important contribution to thymidine selectivity is the strong negative effect of the 2′-hydroxy group as shown through pairwise comparisons of uridine versus 2′-deoxyuridine (δ(ΔG°) = 12.1 kJ/mol) and of 5-methyluridine with thymidine (δ(ΔG°) = 11.57 kJ/mol), yielding an average 11.8 kJ/mol. Supporting evidence is provided by the comparison of 5-Br-uridine and 5-I-2′-deoxyuridine (δ(ΔG°) = 12.5 kJ/mol). The total for the 5-Me and 2′-hydroxy interactions thus comes to 11.8 + 4.2 = 16.0 kJ/mol, consistent with the observed difference between thymidine and uridine of 16.1 kJ/mol.

**Figure 4:**
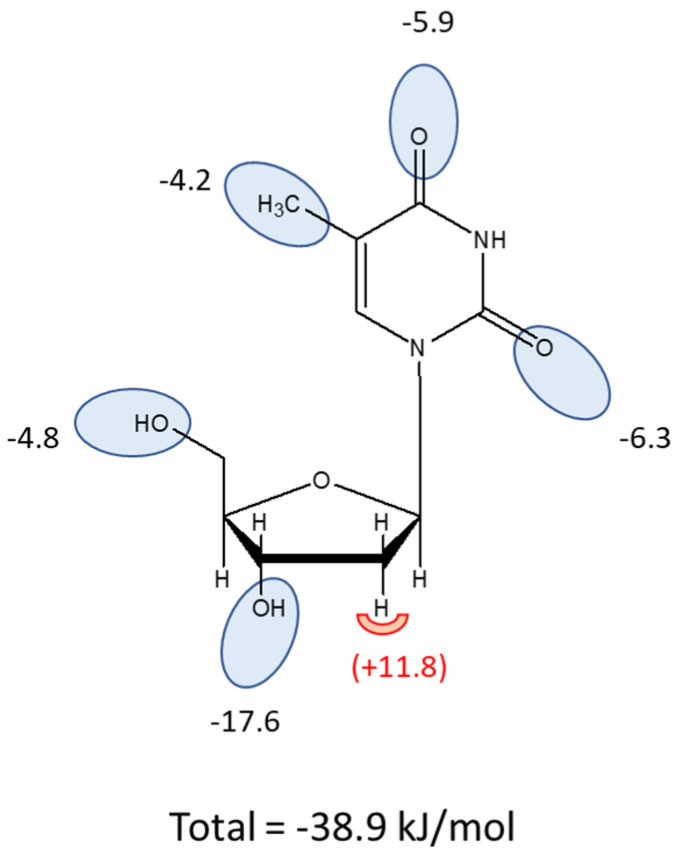
Interaction model for thymidine as a substrate of TcrNT2. Oval shapes indicate putative interactions with the transporter binding pocket, with estimated values for the Gibbs Free energy of the interaction given in kJ/mol. The sum of the proposed interactions is −38.9 kJ/mol. The half-round shape at position 2′ indicates an unfavorable interaction when a hydroxy group is present in this position.

In contrast to the 2′-hydroxy group, the 3′-hydroxy and 5′-hydroxy groups made positive contributions to thymidine binding by TcrNT2, as shown by the following pairwise comparisons: thymidine vs. 3′-deoxythymidine (δ(ΔG°) = −17.6 kJ/mol), of thymidine vs. 5′-deoxythymidine (δ(ΔG°) = −5.26 kJ/mol), and of uridine vs. 5′-deoxyuridine (δ(ΔG°) = −4.41 kJ/mol). For the interaction with the 5′-hydroxy position this yields an average of 4.8 kJ/mol. On the pyrimidine ring, both carbonyl groups also made a positive contribution to thymidine binding, as evidenced by the comparisons of thymidine with 2-thiothymidine and 4-thiothymidine (δ(ΔG°) = −6.33 and −5.86 kJ/mol, respectively). Altogether, this yields a model where TcrNT2 interacts positively with 5-methyl, 2-oxo, 4-oxo, 3′-hydroxy and 5′-hydroxy while a 2′-hydroxy group prevents high affinity binding, through steric or electrostatic effects (Figure 4). The sum of the interactions depicted in Figure 4 is 38.9 kJ/mol, close to the experimental value of −37.5 kJ/mol derived from the experimental K_m_ value (Table 2).

### 2.6. Evaluation of Fluorinated Pyrimidines against T. cruzi

It was shown that the structural analogue of thymidine 5-F-2′-deoxyuridine is a substrate of TcrNT2 when this transporter is heterologously expressed in a *Leishmania* Cas9^ΔNT1^ strain. However, the expression of TcrNT2 did not result in the *Leishmania* parasites becoming sensitized to 5-F-uridine and 5-F-uracil, which could be due to innate insensitivity of *L. mexicana* promastigotes rather than issues of drug transport. Therefore, the effect of the fluorinated pyrimidines 5-F-2’-deoxyuridine, 5-F-uracil and 5-F-uridine was assessed in *T. cruzi*. As a first proxy to analyze the anti-*T. cruzi* effect of these compounds, epimastigotes were incubated in the presence of different concentrations of the three compounds and their growth was monitored. For comparisons, epimastigotes were also incubated with the vehicles used to dissolve the drugs, PBS for 5-F-2′-deoxyuridine and 5-F-uracil, and DMSO for 5-F-uridine (negative controls). As an additional control, proliferation assays were performed in the presence of different concentrations of benznidazole, the most widely used drug for treating CD. The positive control for antiproliferative activity consisted of parasites incubated with a combination of 60 μM rotenone and 0.5 μM antimycin.

**Figure 5:**
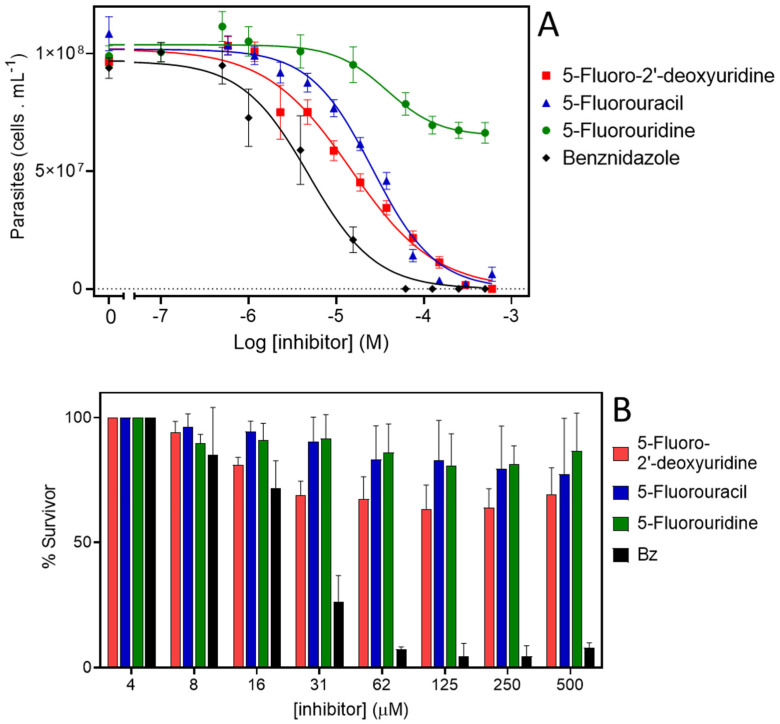
Activity of 5-F-pyrimidines against *T. cruzi*. (**A**). Dose response curves of epimastigotes treated with 5-F-2′-deoxyuridine, 5-F-uracil, 5-F-uridine, or benznidazole. Exponentially proliferating epimastigotes were treated with different concentrations of drugs. The parasite proliferation at the middle-exponential proliferation phase was recorded and used to fit a dose–response sigmoidal function. (**B**). Cell-culture derived trypomastigotes were incubated with different concentrations of 5-F-2′-deoxyuridine, 5-F-uracil, 5-F-uridine, or benznidazole, and their viability was assessed by counting motile parasites in a Neubauer chamber.

The proliferation of the epimastigotes was followed for all treatments, for up to nine days (Supplemental Figure S8). Data on cell densities at the 5th day of proliferation were used to calculate the IC_50_ for each compound. The epimastigotes were insensitive to 5-F-uridine at concentrations up to 500 µM. However, we detected a trypanocidal activity with an IC_50_ of 16.8 ± 0.01 µM for 5-F-2′-deoxyuridine, and 25.9 ± 0.01 µM for 5-F-uracil (Figure 5A). The latter observation indicates the possible presence of a still unidentified uracil transporter in these parasites, similar to the situation in other kinetoplastids where uracil-specific transporters were identified [44,45] and apparently not encoded by an ENT-family gene [46]. The parasites were verified to be fully sensitive to benznidazole, with an IC_50_ of 5.19 ± 0. 01 µM, which serves as a quality control in this assay (Figure 5A). As we detected trypanocidal activity against epimastigotes, the toxicity of the compounds was further assessed in the infective trypomastigote stage. As trypomastigotes are non-dividing forms of *T. cruzi*, we incubated the parasites with different concentrations of the compounds for 24 h and the survival was calculated as the percent of motile parasites with respect to the total parasites. Among the analogues, only 2′-deoxy,5-F-uridine presented any activity against this life cycle stage (approximately 30% inhibition of trypomastigotes bursting at 31 μM 5-F-2′-deoxyuridine) (Figure 5B). These results are in agreement with a diminished expression of the ENT-family transporters in this stage, as previously reported [30].

## 3. Discussion

We have previously reported on four *T. cruzi* genes of the ENT family. By expression in *L. mexicana* we determined that TcrNB2 (tritrypdb TcCLB.506773.50) is a specific carrier for adenine [31]. We also identified TcrNB1 (TcCLB.511051.30) as a hypoxanthine/guanine transporter, TcrNT1 (TcCLB508645.40) as an inosine guanosine transporter and TcrNT2 (TcCLB.506445.110) as a high affinity thymidine carrier with relatively low affinity for uridine that was not inhibited by purine or pyrimidine nucleobases at 1 mM [30]. The discovery that some tubercidin analogues displayed highly promising levels of activity against *T. cruzi* [16,19,20] prompted us to try to identify the *T. cruzi* transporter for tubercidin.

Thymidine transport had previously been reported to be deficient in a tubercidin-resistant strain of *T. cruzi* [29] but our preliminary characterization of the TcrNT2 thymidine transporter had only shown low affinity for this nucleoside antibiotic (K_i_ = 700 ± 110 µM). Here, we express TcrNT2 in *L. mexicana* from which the only pyrimidine nucleoside transporter locus, NT1, has been deleted, creating a near-*null* background for thymidine uptake that is slightly superior to the expression conditions in *T. brucei*, which retained multiple copies of a P1-type nucleoside transporter capable of transporting thymidine, albeit with low affinity [47]. TcrNT2-mediated thymidine transport in Cas9^ΔNT1+TcrNT2^ yielded very similar affinities as previously obtained in *T. brucei*, with K_m_ values of 0.26 ± 0.05 and 0.22 ± 0.01 µM, respectively, as well as virtually identical K_i_ values for 2′-deoxyuridine (1.31 ± 0.002 and 1.11 ± 0.10 µM, respectively), confirming the validity of the expression system. The low affinity for tubercidin (K_i_ = 852 ± 2 µM) was likewise confirmed, and 7-bromo-tubercidin TH1003 displayed similarly low affinity (K_i_ = 781 ± 41 µM).

We next attempted to understand the binding pose of thymidine that gave it its high level of selectivity over other pyrimidine nucleosides, following procedures previously employed for other protozoan and human transporters [27,28,43,45,48,49,50]. Both carbonyl groups appear to be involved in binding interactions as 2-thiothymidine and 4-thiothymidine both displayed ~6 kJ/mol lower ΔG° of binding relative to thymidine. This can partially explain the selectivity over cytidine (K_i_ = 728 ± 71 µM [30]), which has an amine group, i.e., a hydrogen bond donor, rather than an oxo group (H-bond acceptor) on position 4. A further group on the pyrimidine ring that is contributing to binding is the methyl on position 5, which is consistently positive by pairwise comparisons (Tmd vs. 2′-dUrd; Urd vs. 5-Me-Urd). Iodination at position 5 also yields a similar binding advantage, of −4.8 kJ/mol (2′-dUrd vs. 5-I-2′-dUrd), as does bromination (Urd vs. 5-Br-Urd; −4.3 kJ/mol) while fluorination does not significantly change the binding affinity (*p* > 0.05). For the ribose ring, the absence of a 2′-hydroxy is essential (compare Tmd vs. 5-Me-Urd, 11.6 kJ; Urd vs. 2′-dUrd, 12.1 kJ/mol). Together, the presence of 2′-hydroxy and the absence of 4-oxo and 5-Me suffice to explain the low affinity of cytidine, as well as the intermediate affinity of uridine. The absence of binding of nucleobases can be attributed to the contributions of the 3′ and 5′ hydroxy groups. The interaction of TcrNT2 with the 3′-hydroxy group is particularly strong at −17.6 kJ/mol, compared with an average estimate of −4.8 kJ/mol for 5′-hydroxy. The sum of the estimated interactions with the individual groups is close to the binding energy calculated for thymidine from the K_m_ (−38.9 vs. −37.5 kJ/mol), validating the model presented in Figure 4. This binding mode shares the proposed hydrogen bonds with the 3′ and 5′ hydroxy groups proposed for binding pyrimidine nucleosides by the *T. brucei* P1 and *T. gondii* AT2 transporters [50], but the interactions with the pyrimidine ring are different. TgAT2 does not discriminate between thymidine and uridine and is thus indifferent to the 5-methyl group, nor does it bind 2-thio and 4-thio-uridine with lower affinity than uridine, ruling out interactions with the oxo groups as well. Instead, the author proposed a strong interaction of TgAT2 with N3(H), in addition to interactions of the π-electrons of the pyrimidine ring.

The preference of TcrNT2 for 5-F-2′-deoxyuridine over 5-F-uridine or 5-F-uracil that follows from the binding model mirrored the relative activity of the 5-fluorinated pyrimidines against *T. cruzi* epimastigotes, although this should not be ascribed only to the rate of transport through TcrNT2. For instance, 5-F-uracil was only ~2-fold less active than 5-F-2′-deoxyuridine although nucleobases including uracil have no measurable affinity for TcrNT2 at all [30]. Our survey of *T. cruzi* ENT family transporters did not identify a uracil transporter, but this is not surprising as in both *Leishmania* spp. [45] and *T. brucei* [38,44] uracil transport activities have been characterized, but were apparently not encoded by ENT family genes [46]. Thus, further studies of uracil and 5-F-uracil uptake will have to be performed with *T. cruzi* epimastigotes.

Despite being a very good substrate of TcrNT2, 5-F-2′-deoxyuridine had little effect against trypomastigotes, perhaps because the transporter is expressed to a much higher extent in intracellular amastigotes and in insect-form epimastigotes [30], i.e., in the rapidly dividing life cycle stages. Even so, 5-F-2′-deoxyuridine monophosphate is a well-known inhibitor of thymidylate synthesis (TS) [51] and thus acts on the same pathway as the dihydrofolate reductase (DHFR) inhibitor trimetrexate, which displays mid-nanomolar activity against both trypomastigotes and amastigotes [52]. Indeed, in kinetoplastids the two enzymes form a single bifunctional protein DHFR-TS [53] and 5-F-2′-deoxyuridine is quite active against *Leishmania major*, *L. mexicana* [36] and bloodstream form *T. brucei* [47,54], with EC_50_ values of 1.4 µM, 1.7 µM and 5.2 µM, respectively. The most likely explanation for the lack of activity of fluorinated pyrimidines 5-F-uracil and 5-F-2′-deoxyuridine seems to be that they are not effectively metabolized to the deoxyribonucleotide monophosphate 5-F-dUMP that is the active form of these drugs, although this could be readily detected by metabolomics in *L. major*, *L. mexicana* and *T. brucei* after exposure to 5-F-2′-deoxyuridine [36,47].

Despite the low affinity of TcrNT2 for tubercidin, the drug was clearly a substrate for this transporter, as its expression in Cas9^ΔNT1^ sensitized this cell line by over 30-fold (*p* < 0.001; Table 1). Compounds with any 7-halogen substitution retained a large, highly significant shift in the EC_50_ value, notably 41.9-fold for 7-F-tubercidin FH3167. The 7-ethynyl analog FH3143 was associated with a smaller shift, but it should be noted that the Cas9^ΔNT1^ line had retained high sensitivity (EC_50_ 0.93 µM) to it; for the same reason, 7-(trifluoromethyl)-tubercidin FH6367 did not show a significantly lower EC_50_ for Cas9^ΔNT1+TcrNT2^. The 7-phenyl analog TH1004 was also a substrate (*p* < 0.05) but as TcrNT2 failed to restore the sensitivity to close to that of the control *L. mexicana* Cas9 strain, it would appear to be a relatively poor substrate. In contrast, 7-(3,4-chlorophenyl)-tubercidin FH3147 appeared to be a very good substrate and the EC_50_ of Cas9 and Cas9^ΔNT1+TcrNT2^ was nearly identical (8.41 ± 1.61 and 13.7 ± 1.25 µM, respectively, *p* > 0.05) although the compound had no effect on the NT1-KO strain at 100 µM. The at best modest shifts in the EC_50_ values of 7-halogen-3′-deoxy- or 7-ethynyl-3′-deoxytubercidins could indicate that the 3′-hydroxy of 7-substituted is important for their transport, just as it is for thymidine, but the small shift could as easily be related to the lack of resistance in the NT1-KO strain. Moreover, 7-(3,4-dichlorophenyl)-3′-deoxytubercidin FH8513 was more clearly a substrate of TcrNT2 (3-fold shift in EC_50_, *p* < 0.001) and 3′-F-tubercidins appeared to be good substrates, particularly 7-I-3′-F,3′-deoxytubercidin JB5226 (EC_50_ shift 11.6-fold, *p* < 0.001). Thus, we conclude that the 3′-hydroxy of the 7-substituted tubercidins is likely not essential for recognition by TcrNT2 and that their ribose moieties may be differentially orientated from that of thymidine, as recently described for the binding of adenosine and oxopurine nucleosides by the *T. gondii* transporter Tg244440 [49]. It was not possible to conduct a full study of tubercidin binding by TcrNT2 owing to the low affinity and the limits of solubility.

In summary, we have performed a full characterization of the *T. cruzi* NT2 thymidine transporter and determined its binding mode and affinity for potential antimetabolites including fluorinated pyrimidines such as 5-F-2′-deoxyuridine, which were also evaluated for activity against epimastigotes and trypomastigotes. Tubercidin and a range of 7-substituted analogs displayed significantly lower EC_50_ values to a cell line expressing TcrNT2 than the control cells lacking this activity, showing that these antimetabolites are substrates for this transporter.

## 4. Materials and Methods

### 4.1. Parasite Strains and Cultures

We used *L. mexicana* promastigotes of the Cas9 strain developed by Beneke at al. [32] to generate the ΔNT1 strain, and from there the Cas9^ΔNT1+TcrNT2^ (see below); The original Cas9 strain was generously donated by Prof. Eva Gluenz (University of Bern, Switzerland). All strains in standard HOMEM (GIBCO, Life Technologies, Paisley, UK) supplemented with 10% heat-inactivated fetal bovine serum (FBS; PAA Laboratories, Linz, Austria) and 1% of a penicillin–streptomycin solution (10,000 U mL^−1^ each; Life Technologies) at 25 °C, as described [48].

*T. cruzi* epimastigotes (CL Brener strain) were maintained in exponential proliferation by subculturing the parasites every 48 h in Liver Infusion Tryptose (LIT) medium at 28 °C [55]. Trypomastigotes were obtained by infecting CHO-K_1_ cells. CHO-K_1_ cells were cultivated in RPMI-1640 medium supplemented with 10% heat-inactivated fetal calf serum (FCS), 0.15% (*w/v*) NaH_2_CO_3_, 100 µg mL^−1^ penicillin and 130 µg mL^−1^ streptomycin and incubated at 37 °C in a humidified atmosphere containing 5% CO_2_. CHO-K_1_ cells were initially incubated with metacyclic trypomastigotes obtained by in vitro differentiation of epimastigotes as previously described [56] (multiplicity of infection: 50 parasites per cell) for 4 h. After washing out the non-internalized parasites, the cells were incubated overnight in RPMI medium supplemented with 10% FCS at 37 °C and then maintained at 33 °C in a humidified atmosphere containing 5% CO_2_ as previously reported [57]. Infected cells were maintained at 33 °C in the presence of 10% FCS. Infected cell-derived trypomastigotes started to burst into the culture medium at the 5th day post-infection. Trypomastigotes collected at day 5 or 6 post infection were used for the viability assays.

### 4.2. Transport Assays

Transport assays were conducted with radiolabeled nucleosides [*methyl*-^3^H]-thymidine (20 Ci/mmol; PerkinElmer (Waltham, MA, USA)) and [2,8-^3^H]-adenosine (40 Ci/mmol; American Radiolabeled Chemicals (ARC, St-Louis, MO, USA)). Cells were harvested from mid-log phase cultures by centrifugation and washed into an assay buffer (AB) as described [58], at a density of 1 × 10^8^ cells mL^−1^. Transport assays were then performed exactly as described previously, using an oil-stop protocol [43,59]. Briefly, 100 µL of cell suspension (10^7^ cells) was mixed with 100 µL of [^3^H]-substrate at 2 × final concentration and incubated for a pre-set time prior to the addition of 800 µL of ice-cold AB containing a saturating concentration of unlabeled permeant (usually 1 or 2.5 mM) and immediate centrifugation through an oil layer to separate cells from extracellular radiolabel. The 100 µL of [^3^H]-substrate could also contain unlabeled substrate or an inhibitor at 2 × concentration, as appropriate. Next, the microfuge tubes were flash frozen in liquid nitrogen; the tips with the cell pellets cut off and collected in scintillation tubes. Cells were solubilized by incubation in 2% SDS under gentle shaking on a rocking platform prior to the addition of scintillation fluid (Scintilogic U, Lablogic, Sheffield, UK). The tubes stored in the dark overnight before counting in a 300SL Hidex Scintillation Counter (Lablogic).

Michaelis-Menten constants (K_m_) were calculated by plotting transport rates of [^3^H]-substrate against the concentration, using the Michaelis-Menten equation V_0_ = V_max_([substrate]/([substrate] + K_m_)) and non-linear regression in GraphPad Prism 8. Inhibition constants (K_i_) were calculated using the Cheng-Prusoff equation: K_i_ = IC_50_/(1 + (L/K_m_)), where L represents the radiolabel concentration [60]. The IC_50_ was obtained by a non-linear regression of the inhibition curve of transport rate versus inhibitor concentration, using an equation for a sigmoid curve with variable slope (GraphPad Prism 8). The Gibbs free energy ΔG° was obtained using the equation ΔG° = −RTln(K_i_), in which R is the gas constant and T is the absolute temperature. As discussed previously [43], these equations apply to competitive rather than non-competitive inhibition. Given that only close analogues of the substrate were used as potential inhibitors and that Hill slopes were consistently close to −1, it is very likely that inhibition was indeed competitive.

### 4.3. Growth Curves

The growth rates of the *L. mexicana* promastigotes were determined in the standard HOMEM medium supplemented with 10% FBS. After every 24 h, cells were counted in a sample of the culture, using either a Neubauer hemocytometer chamber (Hawksley, UK) or by a coulter particle counter and size analyzer (Beckman, Indianapolis, IN, USA) to count the cells in triplicate. An average of the triplicate readings was taken and plotted using GraphPad Prism 8 software to obtain the growth curves.

### 4.4. Molecular Techniques

The sequences of the nucleotide and amino acid for a gene of interest were obtained from GeneDB (genedb.org) and TritrypDB (tritrypdb.org/tritrypdb) websites. The sequence alignments and the primers that were used in this project were created and designed by using the CLC Genomics Workbench version 7.0 software package (CLC bio, Qiagen, Aarhus, Denmark).

All PCR primers used in this project were synthesized by Eurofins MWG Operon (Ebersberg, Germany) or Sigma-Aldrich (Dorset, UK). PCR with specific primers was used to amplify the genomic DNA of *L. mexicana*, or *T. cruzi* DNA from a plasmid. In order to determine the best annealing temperature, a gradient PCR was performed. DNA for expression constructs was amplified by the Phusion High-Fidelity DNA Polymerase, whereas GoTaq DNA Polymerase was used for the PCR screening. Upon completion of the PCR reaction, a 1% or 2% agarose gel (1 g or 2 g of agarose in 100 mL of 1% TAE buffer) was run to visualize the PCR products with 5 μL of SYBR Safe DNA gel stain (Invitrogen) under UV light. *L. mexicana* genomic DNA was extracted using the NucleoSpin Tissue kit (MACHEREY-NAGEL, Germany), according to the manufacturer’s instructions. The concentration of DNA was measured by using the NanoDrop-1000 spectrophotometer (Thermo Scientific, UK), and the specimens of the DNA were kept at a temperature of −20 °C.

### 4.5. Generation of Plasmids for Expression and Transfection into L. mexicana Promastigotes

The pNUS-HcN plasmid was used to express the *T. cruzi* thymidine transporter TcCLB.506773.50 (TcrNT2) in the *L. mexicana* Cas9^ΔNT1^ strain. The appropriate restriction enzymes were used to digest the pNUS-HcN plasmid and TcrNT2. The T4 DNA Ligase kit was used to ligate the digested gene into pNUS-HcN, which was transformed into XL1-blue *E. coli* cells by heat shock. Colonies were subjected to PCR screening using the forward primer of TcrNT2 (HDK1551) and the reverse primer of pNUS-HcN plasmid (HDK340), which was used to detect colonies having the desired target gene. Correct sequence in a number of positive colonies was verified by Sanger sequencing (Source Bioscience, Livingston, UK).

### 4.6. Targeted CRISPR Gene Knockout Plasmids

The knockout strategy of the *L. mexicana* NT1 locus, containing the two nucleoside transporter genes (*NT1.1* and *NT1.2*), was performed exactly as described by Beneke et al. [32]. The necessary primers (G00, HDK1502; 3′-sgRNA, HDK1508; 5′-sgRNA, HDK1510) were designed using the LeishGEdit online platform [33] and are listed in Supplemental Table S1. To create CRISPR plasmids specific to the target locus of *NT1*, pTBlast and pTPuro plasmids (with blasticidin and puromycin cassettes, respectively) were amplified using primers HDK1507 and HDK1509 (Table S1). These constructs were to integrate into the *NT1* locus after cutting with the two sgRNAs.

The two sgRNA templates and two resistance cassettes (pTBlast and pTPuro) were transfected into *L. mexicana* Cas9 as described by Beneke et al. [32] into *L. mexicana* Cas9. Briefly, the two sgRNA templates and two knockout resistance cassettes were heat-sterilized at 94 °C for 5 min. Then, 1 × 10^7^ cells of *L. mexicana* Cas9 promastigotes were washed with 150 µL of transfection buffer and mixed with 100 µL of the heat-sterilized mixture of the two sgRNA templates and the two KO resistance cassettes. The mix was electroporated with an Amaxa Nucleofector (Amaxa AG, Cologne, Germany), using Program X-001. Cells were then transferred to 20 mL HOMEM medium containing 10% FBS and allowed to recover overnight at 25 °C. After recovery, 5 μg mL^−1^ of blasticidin and 20 μg mL^−1^ of puromycin were added to the culture as a selective marker for the knockout constructs, and the cells were plated out in the 96-well plate to produce individual clones by limiting dilution (1:10, 1:25 and 1:100).

### 4.7. Quantitative Real-Time PCR (qRT-PCR)

qRT-PCR was carried out as previously described [54] to ascertain the level of expression of TcrNT2 in Cas9^ΔNT1+TcrNT2^ compared to the Cas9 control cells. RNA was extracted using the NucleoSpin RNA kit (Macherey-Nagel, Düren, Germany) in accordance with the manufacturer’s instructions. A NanoDrop ND-1000 spectrophotometer was used to quantify the RNA concentration and samples were stored at a temperature of −80 °C until use. qRT-PCR primers were designed using Primer Express 3.0 software. cDNA was synthesized using the Precision nanoScriptTM 2 Reverse Transcription kit (PrimerDesign Ltd., Camberley, UK). Expression was normalised to housekeeping gene GPI8, which is a standard reference gene in *L. mexicana* [61]. For each primer pair the primer efficiency was measured by the Pfaffl method [62]. The cDNA was amplified using the PrecisionPLUS OneStep RT-qPCR Master Mix kit (PrimerDesign Ltd., Camberley, UK) in a 7500 Real-Time PCR System coupled to a desktop computer (Thermo Fisher Scientific, Altrincham, UK). To ensure that only one product at a time was amplified, a dissociation curve was used. Samples without reverse transcriptase (RT) or cDNA were used in the experiment as negative controls. Relative quantification was calculated using the delta delta ct method. Applied Biosystems 7500 Fast Real-Time PCR System Software (Thermo Fisher Scientific, Altrincham, UK) was used for analysis of data. Each experiment was carried out with three independent determinations.

### 4.8. Drug Sensitivity Assays Using Alamar Blue Dye

The Alamar blue assay (resazurin sodium salt) was used to determine the drug sensitivity of the *L. mexicana* promastigotes in vitro. The assay is based on the reduction of resazurin sodium salt (blue and non-fluorescent) to resorufin (pink color and fluorescent) by live cells [63]. HOMEM medium with 10% FBS (100 μL) was added to all wells of a white 96-well plate apart from the first well. 200 μL of a known concentration of test compounds was diluted in HOMEM medium and placed in the first well. Compounds were tested in one plate and every compound was diluted over two rows (23 concentrations), leaving the last well of every dilution as a negative control having 100 μL HOMEM medium. Next, 100 μL of *L. mexicana* culture at a density of 2 × 10^6^ cells mL^−1^ was added into every well in the plate and incubated for 72 h at a temperature of 25 °C after which resazurin sodium salt (Sigma) (20 μL of a solution of 12.5 mg in 100 mL PBS) was added and the plate was incubated under the same conditions for a further 48 h. Fluorescence was measured in a FLUOstar OPTIMA plate reader (BMG Labtech, Germany), at a wavelength of 544 nm for excitation and 620 nm for emission. The EC_50_ values and fluorescence data were determined and plotted by the GraphPad Prism 8 Software using an equation for a sigmoid curve with variable slope. Pentamidine was used as positive control and every experiment was performed 3–4 times independently.

### 4.9. In vitro Inhibition of Proliferation Assays on Epimastigotes

The density of exponentially proliferating epimastigotes for each assay was adjusted to 2.0 × 10^6^ parasites mL^−1^, transferred into 96-well plates (200 μL/well) and incubated with different concentrations of the compounds 5-F-2′-deoxyuridine, 5-F-uracil, 5-F-uridine or benznidazole. A combination of 60 μM rotenone and 0.5 μM antimycin (RA) was used as a positive control for proliferation inhibition (positive control), as previously described [64]. Untreated parasites were incubated in the presence of the vehicle used to dilute the compounds (PBS or DMSO) (negative controls for the anti-*T. cruzi* activity). The epimastigote proliferation was measured as previously reported, by reading the optical density (OD) at 620 nm every 24 h for 9 days (which collects readings through the exponential and stationary phases) [65]. The OD values were converted to cell density values (parasites per mL) by using a calibration curve obtained by measuring the OD values at 620 nm of parasite suspensions at different known densities [64]. The half-maximal inhibitory concentrations (IC_50_) were determined during the exponential growth phase (fifth day) by adjusting a sigmoidal dose-response function to experimentally obtained data using GraphPad Prism 8. The compounds were assessed in quadruplicate in each experiment, and the results correspond to four independent experiments.

### 4.10. Effect of Compounds on Cell Derived Trypomastigotes

To measure the effect of compounds, cell derived trypomastigotes were collected from the extracellular medium at the fifth or sixth day after infection and counted in a Neubauer chamber [65]. A total of 1 × 10^5^ trypomastigotes/well in RPMI medium supplemented with 10% FCS were seeded in 96 well plates. Different concentrations of compounds or benznidazole (as a control) were added to the plates and were incubated for 24 h at 37 °C [66]. After incubation the trypanocidal effect was assessed by counting the percent of motile parasites in a Neubauer chamber. Sigmoidal dose-response function was fitted to data using GraphPad Prism 8. All experiments were performed in triplicate and data correspond to the mean values obtained from three independent experiments.

## Figures and Tables

**Table 1:** The EC_50_ of different adenosine analogues and 5-F-2′-deoxyuridine on *L. mexicana* Cas9*, L. mexicana* Cas9^ΔNT1^ and *L. mexicana* Cas9^ΔNT1+TcrNT2^ promastigotes, obtained from drug sensitivity assay.

	Cas9	*L. mexicana* Cas9^ΔNT1^			*L. mexicana* Cas9^ΔNT1+TcrNT2^			Structure
	EC_50_ (µM) AVG ± SEM	EC_50_ (µM) AVG ± SEM	RF vs. Cas9	*p* Value vs. Cas9	EC_50_ (µM) AVG ± SEM	RF vs. Δ*NT1*	*p* Value vs. Δ*NT1*	
5-F-2′-dUrd	2.19 ± 0.11	58.4 ± 8.7	26.6	*p <* 0.01	3.94 ± 0.36	14.8	*p <* 0.01	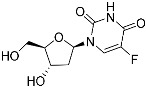
Tubercidin *	1.02 ± 0.16	25.7 ± 0.94	25.2	*p <* 0.001	0.82 ± 0.13	31.2	*p <* 0.001	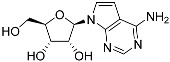
FH3167	0.22 ± 0.09	8.95 ± 1.59	40.7	*p <* 0.01	0.21 ± 0.02	41.9	*p <* 0.01	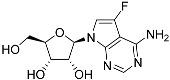
FH3169 *	0.24 ± 0.055	2.00 ± 0.19	8.43	*p <* 0.01	0.32 ± 0.05	6.26	*p <* 0.01	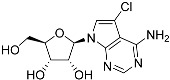
TH1003	0.16 ± 0.018	2.90 ± 0.57	18.4	*p <* 0.01	0.38 ± 0.02	7.68	*p <* 0.05	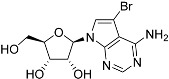
FH3141	1.08 ± 0.202	14.5 ± 1.77	13.4	*p <* 0.01	1.86 ± 0.21	7.83	*p <* 0.01	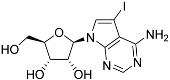
FH6367	1.42 ± 0.177	2.05 ± 0.42	1.44	*p* > 0.05	1.07 ± 0.09	1.91	*p* > 0.05	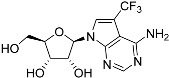
FH3143	0.12 ± 0.021	0.93 ± 0.18	7.74	*p <* 0.05	0.30 ± 0.01	3.11	*p <* 0.05	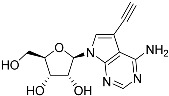
TH1004 *	37.0 ± 0.23	138 ± 10	3.7	*p* < 0.001	101 ± 0.5	1.37	*p* < 0.05	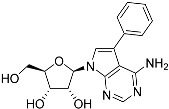
TH1012 *	52.5 ± 0.3	280 ± 18	5.3	*p* < 0.001	96.6 ± 0.3	2.90	*p* < 0.001	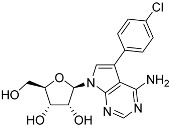
FH3147 *	8.41 ± 1.61	>100 ± 0.00	>11.8	*p <* 0.001	13.7 ± 1.25	7.28	*p <* 0.01	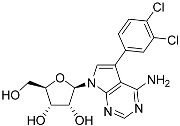
FH10714 *	>500	>500			>500			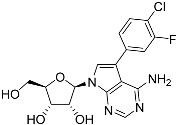
FH8517	3.18 ± 0.78	12.5 ± 1.47	3.93	*p <* 0.01	9.77 ± 0.67	1.28	*p* > 0.05	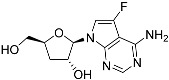
FH7429-UP	8.0 ± 0.8	9.37 ± 1.15	1.17	*p* > 0.05	4.12 ± 0.88	2.27	*p <* 0.05	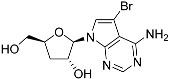
FH8505	47.2 ± 1.47	53.3 ± 5.3	1.13	*p* > 0.05	36.2 ± 1.34	1.47	*p <* 0.05	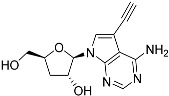
FH8480	>500	>500			>500			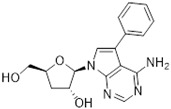
FH8513	~100	93.9 ± 4.0	0.94	*p* > 0.05	31.1 ± 2.8	3.0	*p* < 0.001	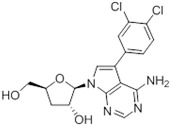
JBMAM030	7.81 ± 0.90	311 ± 13	39.8	*p* < 0.0001	332 ± 1.5	0.94	*p* > 0.05	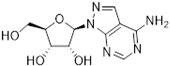
JBMAM021	3.80 ± 0.66	304 ± 23	79.9	*p* < 0.0001	315 ± 6	0.97	*p* > 0.05	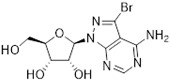
JB602	>500	>500			>500			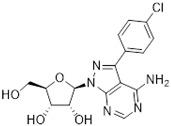
JB588	0.51 ± 0.093	8.82 ± 1.12	17.3	*p <* 0.01	4.10 ± 0.19	2.15	*p <* 0.05	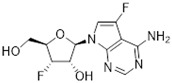
JB526	~100	83.5 ± 8.3	~0.84	*p* > 0.05	7.18 ± 0.31	11.6	*p* < 0.001	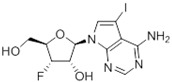
CL5564	>100	>100			17.9 ± 1.4	>5.6		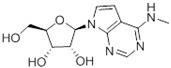
CL4501	~100	84.0 ± 8.1	0.84	*p* > 0.05	37.4 ± 2.4	2.2	*p* < 0.01	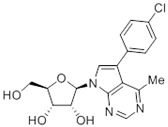
Pentamidine	0.87 ± 0.104	1.25 ± 0.22	1.43	*p* > 0.05	1.12 ± 0.17	1.11	*p* > 0.05	

The EC_50_ are shown as averages in µM (± SEM) of at least 3 independent determinations. Pentamidine is a standard drug used as control in this assay. The *p* values show the level of significance of the sensitivity of the analogues have on the cell lines. The drugs in bold have a significantly different level of sensitivity on the cells. *p* values were determined by unpaired Student’s T-test: Not significant *p* > 0.05; *, values from de Almeida Fiuza et al. [41]. RF: resistance factor in comparison to Cas9 (*L. mexicana* Cas9) and Δ*NT1* (*L. mexicana* Cas9^ΔNT1^); SEM: standard error of mean. 5-F-2′-dUrd: 5-F-2′-deoxyuridine.

**Table 2:** K_i_ for pyrimidine nucleosides and adenosine analogues on the transport of [^3^H]-thymidine by TcrNT2 in *L. mexicana* Cas9^ΔNT1^. IC_50_ values obtained were converted to K_i_ based on the K_m_ of TcrNT2 (average ± SEM). ΔG° represents the change in Gibbs free energy and δ (ΔG°) represents the change in ΔG° of the respective analogues with respect to ΔG° of thymidine.

Inhibitor	K_m_ or K_i_ (μM)	ΔG° (kJ/mol)	δ(ΔG°) (kJ/mol)	*n*
thymidine	0.26 ± 0.05	−37.5	--	3
2-thiothymidine	3.38 ± 0.26	−31.2	−6.33	3
4-thiothymidine	2.79 ± 0.11	−31.7	−5.86	3
Uridine	172 ± 8	−21.5	−16.1	3
5-Br-uridine	30.1 ± 1.8	−25.8	−11.8	3
5-Me-uridine	28.0 ± 0.4	−26.0	−11.6	3
5-F-2′-deoxyuridine	1.50 ± 0.12	−33.2	−4.32	3
5-I-2′-deoxyuridine	0.19 ± 0.04	−38.3	−0.80	3
2′-deoxyuridine	1.31 ± 0.002	−33.6	−3.99	3
3′-deoxythymidine	322 ± 22.6	−19.9	−17.6	3
5′-deoxythymidine	2.20 ± 0.03	−32.3	−5.26	3
5′-deoxyuridine	1021 ± 57	−20.5	−20.5	3
Adenosine ^1^	1560 ± 60	−15.8	−23.2	3
tubercidin	852 ± 2	−17.5	−20.0	2
TH1003	784 ± 41	−19.8	−15.8	2

Value highlighted in bold represents the K_m_ for thymidine. ΔG° was calculated at 25 °C. ^1^ From Campagnaro et al. [30].

## Data Availability

All relevant data are contained in the manuscript and Supplemental Information.

## Supplementary Materials

The following supporting information can be downloaded at: www.mdpi.com/xxx/s1, Figure S1: Sequence and alignment of LmexNT1.1. and LmexNT1.2; Figure S2: PCR amplification of 5’ sgRNA-NT1 and 3’ sgRNA-NT1 templates for knockout of NT1 locus in *L. mexicana* Cas9 and PCR amplification of the blasticidin and puromycin resistance markers; Figure S3: PCR validation of knockout of NT1 region in *L. mexicana* Cas9 by using CRISPR-Cas9 system; Figure S4: Expression levels of NT1 in *L. mexicana* Cas9^ΔNT1^ compared to the control (*L. mexicana* Cas9) determined by qRT-PCR; Figure S5: Restriction digest products for pHDK270 plasmid and confirmation of the presence of *TcrNT2* gene in *L. mexicana* Cas9^ΔNT1^ cells after transfection; Figure S6: The growth of *L. mexicana* Cas9 and Cas9^ΔNT1^ promastigotes and Cas9^ΔNT1^ expressing TcrNT2, in HOMEM medium supplemented with 10% FBS at 25 °C; Figure S7: Sensitivity of various *Leishmania mexicana* strains to 5-F-pyrimidines; Figure S8: Effect of fluorinated pyrimidines on *T. cruzi* epimastigotes. Table S1: List of primers used to generate NT1 KO in *L. mexicana* Cas9.

## Sample Availability

Samples of the compounds can be obtained from the authors subject to availability.
