# Supplementary material for: The Trypanosoma cruzi TcrNT2 Nucleoside Transporter Is a Conduit for the Uptake of 5-F-2′-Deoxyuridine and Tubercidin Analogues
Source: Molecules. 2022 Nov 19;27(22):0. doi: 10.3390/molecules27228045 (PMC9693223; doi:10.3390/molecules27228045)
Supplement: Supplementary file 1 [file molecules-27-08045-s001.zip › molecules-1989544-supplementary.pdf]

Supplemental Figure S1. Sequence and alignment of LmexNT1.1. and LmexNT1.2.

Amino acid sequence of LmexNT1.1 (LmxM.15.1230.1), protein length 491 a. a.

MDTAPDRQDPQEQGESRKWYEMSASEFYVYVVAFMCGVSMMMPINAVFSAPAYIMTYYRYAMQDPDAVPLQ
TNFWNNVMTYYNLIGILVSLIMEPLTLLSWFRRIPIKARLFGGLIILIVEIIVLMVVPARGTNEAGAVATICCASFIGGF
GKSIFESTTYGMFGAFPSSFTSTMMGGVGMSGVLTSLLQISVKAALPDSYEGVKKQSKIYYGLDVGIQIMTFIALGLL
RFNSFAQNYFGDLGAVKSKVDAGKLSPEVLCNPDERPVHGKEGRNSSSGKVVPALGEVQMVTAKSESPDAAEEAS
WPQEVEGPTSNEILVATSIFSTLRCIKWMFIACAFDFLITLFLFPAIAVGMFPDSKWFSTIAVFIFNVFDVLGRFSPSLK
FMWPKTYKQRWIIVAASFARVIFVPLLLLHSYHYIPSEEYGYVMEVIFGFSNGYVGSMALVLGPQSKGIDNDGKRFV
AGTLMGISILVGATIGTVLSIMTQTIRERH

Amino acid sequence of LmexNT1.2 (LmxM.15.1240.1), protein length 491 a. a.

MDTAPDRQDPQDQGESRKWYEMSASEFYVYVVAFMCGVSMMMPINAVFSAPAYIMTYYRYAMQDPDAVPLQ
TNFWNNVMTYYNLIGIVTSLIMEPLTLLSWFRRIPMKARLFGGLIILIVEIIVLMVVPARGTNEAGAVATICITGFIGGF
GKSIFESTTYGMFGAFPSSFTSTMMGGVGMSGVLTSLLQIIVKAALPDSYEGVKKQSKIYYGLDVGIQVMTFIALGLL
RFNSFAQNYFGDLGAVKSKVDAGKLSPEVLCNPDERPVHGKEGRNSSSGKVVPALGEVQMVTAKSESPDAAEEAS
WPQEVEGPTSNEILVATSIFSTLRCIKWMFIACAFDFLITLFLFPGIAVGMFPDSKWFSTIAVFIFNVFDVLGRFSPSLK
FMWPKTYKQRWIIVAASFARVIFVPLLLLHSYHYIPSEEYGYVMEVIFGFSNGYVGSMALVLGPQSKGIDNDGKRFV
AGTLMGISILVGGTIGTVLSIMTQTIRATY

Alignment of LmexNT1.1 (Query) and LmexNT1.2 (Sbjct) using NCBI BLAST two protein sequences

Score	Expect	Method	Identities	Positives
979 bits(2531)	0.0	Compositional matrix adjust.	477/491(97%)	482/491(98%)

```
Gaps

0/491(0%)
```

```
Query  1    MDTAPDRQDPQEQGESRKWYEMSASEFYVYVVAFMCGVSMMMPINAVFSAPAYIMTYYRY  60
            MDTAPDRQDPQ+QGESRKWYEMSASEFYVYVVAFMCGVSMMMPINAVFSAPAYIMTYYRY
Sbjct  7    MDTAPDRQDPQDQGESRKWYEMSASEFYVYVVAFMCGVSMMMPINAVFSAPAYIMTYYRY  66
```

```
Query  61   AMQDPDAVPLQTNFWNNVMTYYNLIGILVSLIMEPLTLLSWFRRIPIKARLFGGLIIILIV  120
            AMQDPDAVPLQTNFWNNVMTYYNLIGI+ SLIMEPLTLLSWFRRIP+KARLFGGLIIILIV
Sbjct  67   AMQDPDAVPLQTNFWNNVMTYYNLIGIVTSLIMEPLTLLSWFRRIPMKARLFGGLIIILIV  126

Query  121  EIIVLMVVPARGTNEAGAVATICCASFIGGFGKSIFESTTYGMFGAFPSSFTSTMMGGVG  180
            EIIVLMVVPARGTNEAGAVATIC   FIGGFGKSIFESTTYGMFGAFPSSFTSTMMGGVG
Sbjct  127  EIIVLMVVPARGTNEAGAVATICITGFIGGFGKSIFESTTYGMFGAFPSSFTSTMMGGVG  186

Query  181  MSGVLTSLLQISVKAALPDSYEGVKKQSKIYYGLDVGIQIMTFIALGLLRFNSFAQNYFG  240
            MSGVLTSLLQI VKAALPDSYEGVKKQSKIYYGLDVGIQ+MTFIALGLLRFNSFAQNYFG
Sbjct  187  MSGVLTSLLQIIVKAALPDSYEGVKKQSKIYYGLDVGIQVMTFIALGLLRFNSFAQNYFG  246

Query  241  DLGAVKSKVDAGKLSPEVLCNPDERPVHGKEGRNSSSGKVVPALGEVQMVTAKSESPDAA  300
            DLGAVKSKVDAGKLSPEVLCNPDERPVHGKEGRNSSSGKVVPALGEVQMVTAKSESPDAA
Sbjct  247  DLGAVKSKVDAGKLSPEVLCNPDERPVHGKEGRNSSSGKVVPALGEVQMVTAKSESPDAA  306

Query  301  EEASWPQEVEGPTSNEILVATSIFSTLRCIKWMFIACAFDFLITLFLFPAIAVGMFPDSK  360
            EEASWPQEVEGPTSNEILVATSIFSTLRCIKWMFIACAFDFLITLFLFP IAVGMFPDSK
Sbjct  307  EEASWPQEVEGPTSNEILVATSIFSTLRCIKWMFIACAFDFLITLFLFPGIAVGMFPDSK  366

Query  361  WFSTIAVFIFNVFDVLGRFSPSLKFMWPKTYKQRWIIVAASFARVIFVPLLLLHSYHYIP  420
            WFSTIAVFIFNVFDVLGRFSPSLKFMWPKTYKQRWIIVAASFARVIFVPLLLLHSYHYIP
Sbjct  367  WFSTIAVFIFNVFDVLGRFSPSLKFMWPKTYKQRWIIVAASFARVIFVPLLLLHSYHYIP  426

Query  421  SEEYGYVMEVIFGFSNGYVGSMALVLGPQSKGIDNDGKRFVAGTLMGISILVGATIGTVL  480
            SEEYGYVMEVIFGFSNGYVGSMALVLGPQSKGIDNDGKRFVAGTLMGISILVG TIGTVL
Sbjct  427  SEEYGYVMEVIFGFSNGYVGSMALVLGPQSKGIDNDGKRFVAGTLMGISILVGGTIGTVL  486

Query  481  SIMTQTIRERH  491
            SIMTQTIR  +
Sbjct  487  SIMTQTIRATY  497
```

Supplemental Figure S2. PCR amplification of 5' sgRNA-NT1 and 3' sgRNA-NT1 templates for knockout of NT1 locus in *L. mexicana* Cas9 and PCR amplification of the blasticidin and puromycin resistance markers.

A) PCR amplification of 5' sgRNA-NT1 and 3' sgRNA-NT1 templates for knockout of *NT1* region (*NT1.1* and *NT1.2* genes) in *L. mexicana* Cas9. L: 100 bp DNA ladder (Promega); lane 1 and 2: 5' sgRNA-NT1 (~120 bp); 3 and 4: 3' sgRNA-NT1 (~120 bp); Negative control (No sgRNA scaffold). B) PCR amplification of blasticidin and puromycin resistance markers. L: 1 kb DNA ladder (Promega); 1 and 2: Blasticidin-NT1 (~1.7 kb); 3 and 4: Puromycin-NT1 (~1.8 kb); Negative control (No genomic DNA).

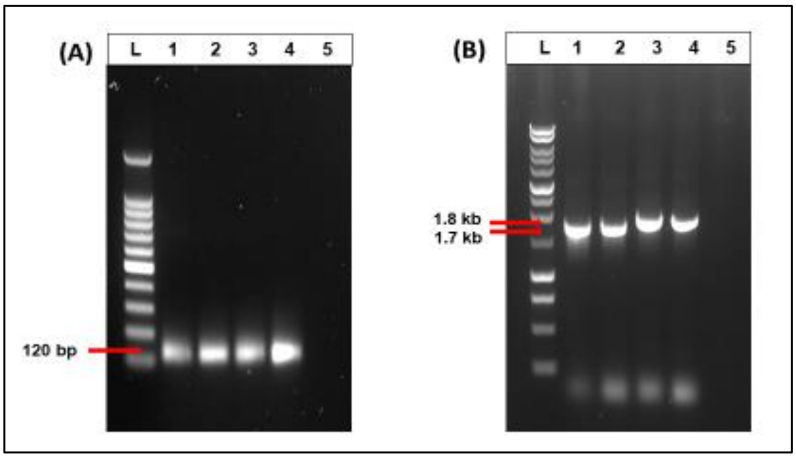

Supplemental Figure S3: PCR validation of knockout of NT1 region in *L. mexicana* Cas9 by using CRISPR-Cas9 system.
L: 1kb DNA Ladder (Promega); 1*: L. mexicana* Cas9; 2: L. mexicana Cas9$^{\Delta NT1}$ 3: Negative control (No genomic DNA).

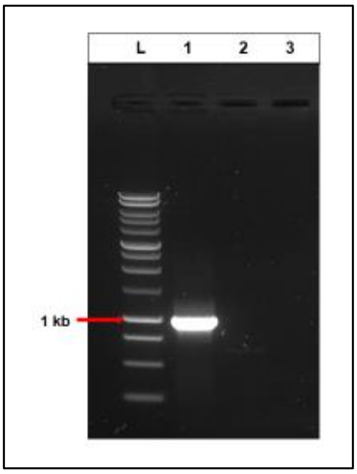

Supplemental Figure S4: Expression levels of NT1 in *L. mexicana* Cas9$^{\Delta NT1}$ compared to the control (*L. mexicana* Cas9) determined by qRT-PCR.

Levels were normalised against the expression level of the housekeeping gene *L. mexicana* GPI8. The presented results are the average of 2 cDNA preparations, each performed in duplicate, and error bars are ± SEM. ***, P < 0.001 by unpaired student's T-test.

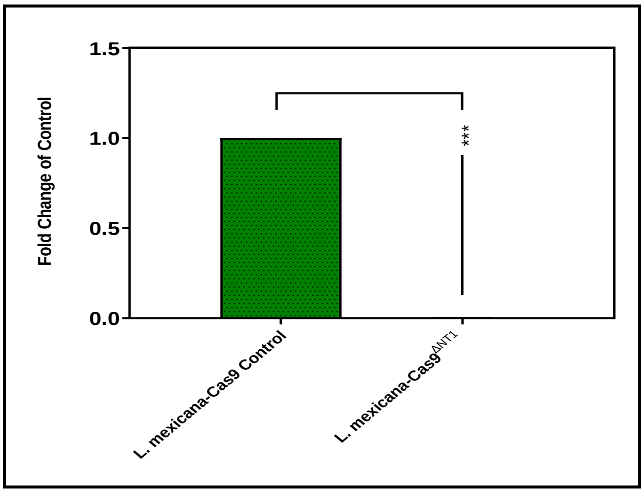

Supplemental Figure S5: Restriction digest products for pHDK270 plasmid and confirmation of the presence of *TcrNT2* gene in *L. mexicana* Cas9$^{\Delta NT1}$ cells after transfection.

A) Restriction digest products for plasmid pHDK270 with *Bgl*II and *Xho*I to release the *TcrNT2* gene. L: 1 kb DNA ladder (Promega); 1: Digested plasmid pHDK270 by *Bgl*II and *Xho*I to release *TcrNT2* (1323 bp) from pHDK270 (~6.2 kb); 2: Undigested plasmid for pHDK270.

B) Confirmation of the presence of *TcrNT2* gene into *L. mexicana* Cas9$^{\Delta NT1}$ strain after transfection by using HDK1551 as forward primer for *TcrNT2* and HDK340 as a reverse primer for pNUS-HcN. L: 1kb DNA Ladder (Promega); 1: Clone 1; 2: Clone 2; 3: Clone 3; 4: Non-transfected control (gDNA from *L. mexicana* Cas9$^{\Delta NT1}$).

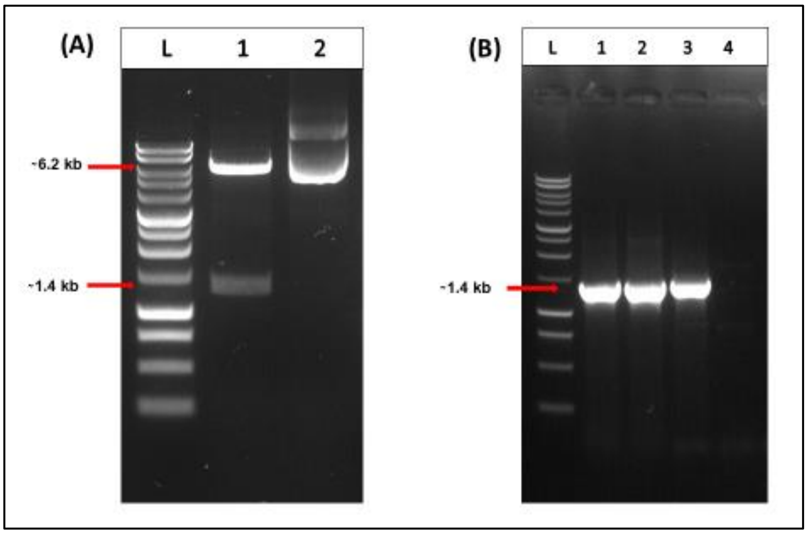

Supplemental Figure S6. The growth of *L. mexicana* Cas9 and Cas9$^{\Delta NT1}$ promastigotes and Cas9$^{\Delta NT1}$ expressing TcrNT2, in HOMEM medium supplemented with 10% FBS at 25 °C.

The cells were seeded at the density of 1×10^5 cells mL^{-1}, and cell densities were determined every 24 h. Each data point in this result represents the mean of two similar independent repeats performed in triplicate.

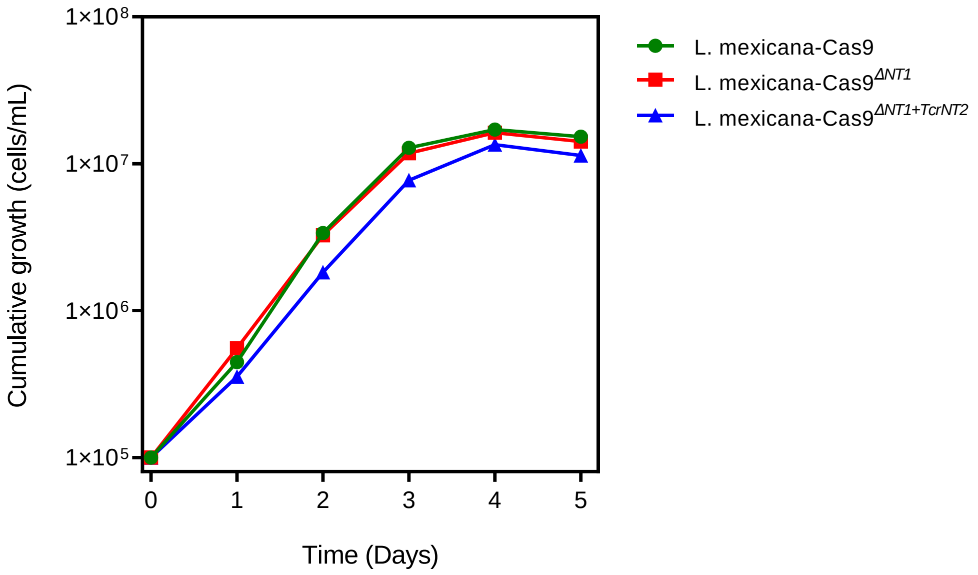

Supplemental Figure S7. Sensitivity of various *Leishmania mexicana* strains to 5-F-pyrimidines.

The EC_{50} values were determined using the Alamar blue (resazurin) method as described in the main paper. Highest concentration in the assay was 500 µM and bars are topped when not achieving 50% reduction in fluorescence at that concentration. Data shown is the average of three independent determinations; error bars are SEM. Pentamidine was used as internal positive control on all plates.

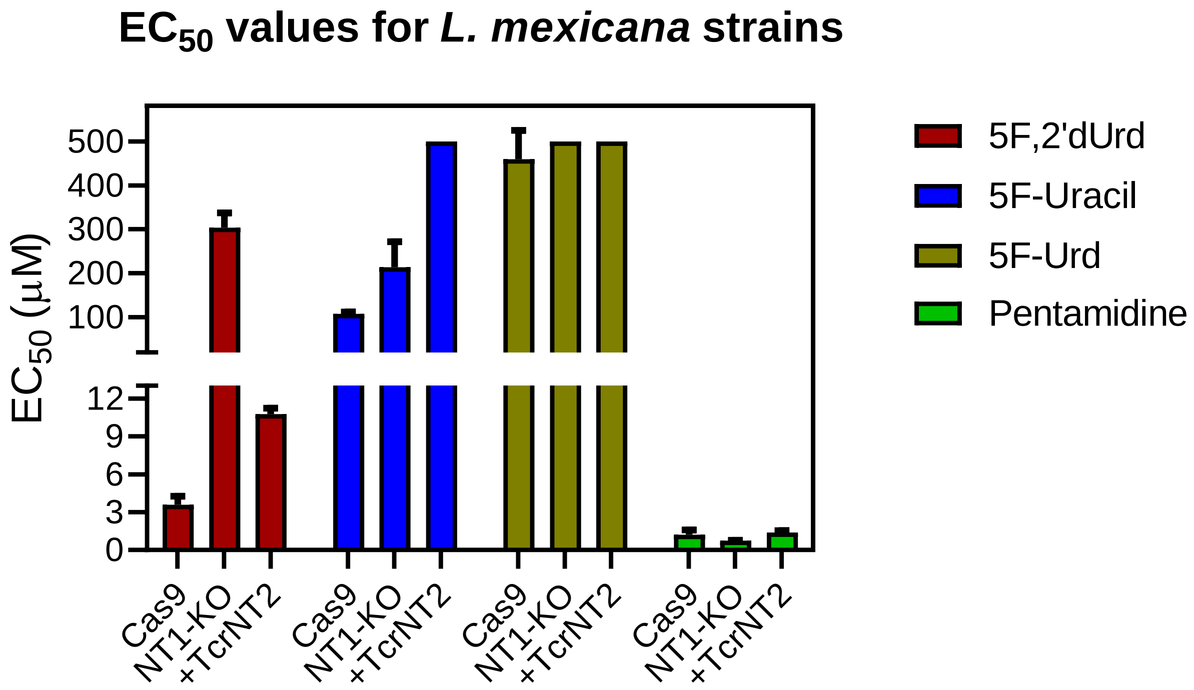

Supplemental Figure S8. Effect of fluorinated pyrimidines on *T. cruzi* epimastigotes.

Proliferation curves of epimastigotes treated with 5-F-2´deoxyuridine, 5-F-uracil, 5-F-uridine, or benznidazole. Exponentially proliferating epimastigotes were cultured in the presence of different concentrations of 2´deoxy-5F-uridine (Panel A), 5F-uracil (Panel B), 5F-uridine (Panel C), or benznidazole Panel D). The parasites were quantified daily. More details in Materials and Methods.

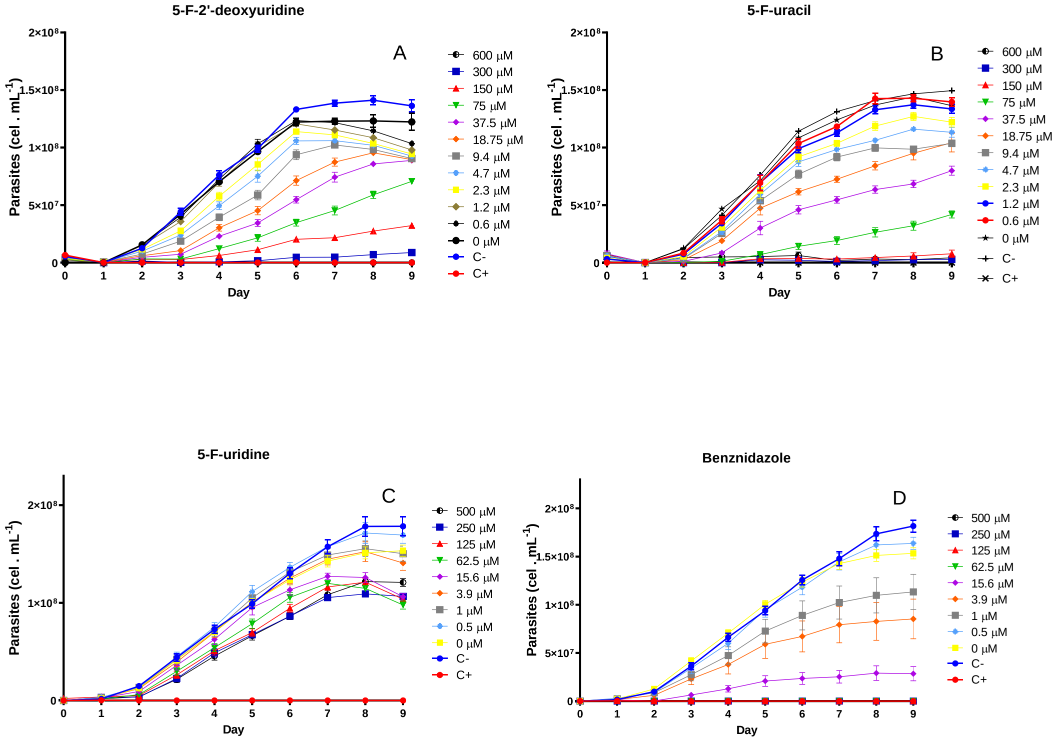

Supplemental Table S1: List of primers used to generate NT1 KO in *L. mexicana* Cas9.

5’ and 3’ sgRNA primers: lower case indicates the T7 RNAP promoter (left), upper case indicates the 20 nucleotides of the sgRNA target sequences (middle) and lower case indicates the Cas9-backbone-start (right).

Upstream forward and downstream primers (NT1 KO): upper case indicates the 30 nucleotides homology flanks for target-gene specific (left) and lower case indicates primer binding sites for pTBlast and pTPuro (right).

	Primer name	Sequence (5’ → 3’)
HDK1502	G00 primer (sgRNA scaffold)	AAAAGCACCGACTCGGTGCCACTTTTTCAAGTTGATAACGGACTAGCCTTATTTTAACTTGCTATTTCTAGCTCTAAAAC
HDK1508	5' sgRNA primer (NT1 KO)	gaaattaatacgactcactataggTGCGACTTTGGATGCACTGAgttttagagctagaaatagc
HDK1510	3' sgRNA primer (NT1 KO)	gaaattaatacgactcactataggACGCATACACAAGCAAGGAGgttttagagctagaaatagc
HDK1507	Upstream forward primer (NT1 KO)	TCGCACACATCTCTCGTCCACAAGGCCCCTgtataatgcagacctgctgc
HDK1509	Downstream reverse primer (NT1 KO)	GCGATCAACAGCAGTGCGCGGGGCACGCACccaatttgagagacctgtgc
HDK1523	Forward primer NT1	TCCGCTGCAAACAAACTTCTGG
HDK1524	Reverse primer NT1	TACGCCGCTACGATGATCCAGC
HDK1551	Forward primer NT1 introducing a *Bgl*II site	AGATCTATGGGACTGGGCTTCGAATTCT
HDK1552	Reverse primer NT1 introducing a *Xho*I site	AAATTTCTCGAGCTACCCGCGCAAGGTCTG
HDK340	Reverse primer pNUS-HcN	CGTGGAGCAGCTGAAGGACA
